# Advanced Maternal Age Differentially Affects Embryonic Tissues with the Most Severe Impact on the Developing Brain

**DOI:** 10.3390/cells12010076

**Published:** 2022-12-24

**Authors:** Caroline Kokorudz, Bethany N. Radford, Wendy Dean, Myriam Hemberger

**Affiliations:** 1 Department of Biochemistry and Molecular Biology, Cumming School of Medicine, University of Calgary, 3330 Hospital Drive NW, Calgary, AB T2N 4N1, Canada; 2 Alberta Children’s Hospital Research Institute, University of Calgary, 3330 Hospital Drive NW, Calgary, AB T2N 4N1, Canada; 3 Department of Cell Biology and Anatomy, Cumming School of Medicine, University of Calgary, 3330 Hospital Drive NW, Calgary, AB T2N 4N1, Canada

**Keywords:** advanced maternal age, embryonic development, transcriptomics, RNA-seq, placenta, brain, heart, facial prominences, uterine stroma, trophoblast stem cells

## Abstract

Advanced maternal age (AMA) poses the single greatest risk to a successful pregnancy. Apart from the impact of AMA on oocyte fitness, aged female mice often display defects in normal placentation. Placental defects in turn are tightly correlated with brain and cardiovascular abnormalities. It therefore follows that placenta, brain and heart development may be particularly susceptible to the impact of AMA. In the current study, we compared global transcriptomes of placentas, brains, hearts, and facial prominences from mid-gestation mouse conceptuses developed in young control (7–13 wks) and aging (43–50 wks) females. We find that AMA increases transcriptional heterogeneity in all tissues, but particularly in fetal brain. Importantly, even overtly normally developed embryos from older females display dramatic expression changes in neurodevelopmental genes. These transcriptomic alterations in the brain are likely induced by defects in placental development. Using trophoblast stem cells (TSCs) as a model, we show that exposure to aging uterine stromal cell-conditioned medium interferes with normal TSC proliferation and causes precocious differentiation, recapitulating many of the defects observed in placentas from aged females. These data highlight the increased risk of AMA on reproductive outcome, with neurodevelopment being the most sensitive to such early perturbations and with potential for lifelong impact.

## 1. Introduction

The single greatest risk factor for a healthy pregnancy is maternal age [1,2,3,4]. The most well-known cause of the increased pregnancy risk due to advanced maternal age (AMA), which in humans is commonly considered age 35 and over, is the exponential increase in chromosome mis-segregations in the oocyte. These germ cell defects often result in pregnancy loss during the first trimester. If the pregnancy is carried to term, these oocyte-inherited chromosomal imbalances lead to gene dosage abnormalities in the offspring, such as—for example—in cases of trisomy 21 [5,6]. Although aneuploidies can be a major cause of fetal demise and congenital defects in the newborn to mothers aged 35 and over, AMA is also associated with a substantial increase in the incidence of serious pregnancy complications that are unrelated to karyotypic abnormalities [7,8]. These complications include later miscarriages, preterm birth, preeclampsia, and low birth weight [1,9,10]. Notably, all these syndromes are frequently linked to a failure in correct placentation unrelated to a decline in oocyte fitness [11]. 

Pregnancy complications with a proven or suspected placental etiology often result in developmental delay and poor fetal growth, deficits that are frequently tied to an insufficient placental nutrient transfer capacity. Moreover, several pregnancy complications have been associated with an elevated prevalence of neurodevelopmental disorders. As such, preeclampsia and preterm birth are correlated with an increased risk of autism spectrum disorders (ASDs) in the offspring [7,12,13,14]. As the incidence of women having pregnancies over the age of 35 has steadily risen to 25% in 2021 in Canada, with similar trends seen elsewhere in the world, complications of pregnancy and their associated impact on the offspring pose increasingly significant problems to the health care system (Statistics Canada. Table 13-10-0416-01 Live births, by age of mother; National Vital Statistics Reports, Vol. 70, No. 17). AMA has also been associated with an increase in birth defects, such as congenital heart anomalies and skull deformations [7,15,16,17].

While the cause of fetal anomalies can be genetically explained in cases of karyotypic imbalances inherited from the oocyte, the underlying mechanisms for the increased frequency of pregnancy complications and congenital defects that lack a chromosomal basis has remained much less explored. To gain insights into this connection, we and others have used the mouse model to study the maternal age-related decline in developmental progression. Similar to the situation in humans, pregnancies to older female mice are associated with a stark increase in developmental problems in the offspring, with a much higher rate of developmental delays, fetal growth retardation, and serious embryonic defects that include cardiac edema, vascular defects, and brain and neural tube closure defects [18,19]. Interestingly, when preimplantation embryos from aged females were transferred into young recipients, they developed largely normally. These data demonstrate that the vast majority of embryonic abnormalities in pregnancies to older female mice are due to age-related maternally induced placentation defects. These defects likely arise as a consequence of declining uterine function that is detrimental for placental development [18]. In line with these findings, a similar experimental rescue of the maternal age-induced increase of congenital heart defects was reported upon ovary transfer from aged into young females, again pointing to the detrimental effects of the aging uterine environment [15].

Placentation defects are known to be closely associated with abnormal heart, brain, and vascular system morphology [20]. Thus, a scenario emerges that links placenta, brain, and cardiovascular development in normal gestation, and finds these organ systems to be at increased risk of developmental failures in pregnancies to aging females. What remains unknown, however, is whether these organ systems are all equally affected by maternal age, or whether specific tissues or cell types are particularly sensitive to maternal age-induced alterations. In this study, we sought to explore the differential impact of maternal age on specific embryonic tissues as well as on the placenta. Using RNA-sequencing to investigate key transcriptional differences between the placenta, heart, brain, and facial prominences, we establish that the embryonic brain is the organ by far the most profoundly affected by AMA. This effect is tied to an insufficient development of the placental trophoblast compartment, which we show occurs as a direct consequence of being exposed to an aging uterine stroma. Collectively, our data demonstrate that AMA causes widespread transcriptomic changes in the developing embryo, with particular emphasis on the brain, even in the absence of gross morphological defects.

## 2. Materials and Methods

### 2.1. Mouse Tissues

All animal work was conducted with approval by the University of Calgary’s animal care committee and with approved animal use protocols in place. All mice used were of the C57Bl/6N (Elite) strain that was procured from Charles River (Wilmington, MA, USA) and housed in the UCalgary’s Health Sciences Animal Resources Centre in IVC cages with ad libitum food and water supply and standard 12-h light-dark cycles. Virgin females were housed until the indicated weeks of age. All stock animals were fed with low fat diet (Pico-vac 5061) to maintain a healthy weight; breeders were on high fat diet (Pico-vac 5062). The intended result of this regime was that young and aged females were of similar weight. The embryos used were produced by inter se matings, counting the day of the vaginal plug as embryonic day (E)10.5. All animal work was conducted with approval by the University of Calgary’s animal care committee, and with appropriate Health Sciences Animal Care Committee (HSACC)-approved animal use protocols in place (protocol numbers AC18-0177 and AC18-0191). The approval dates of these protocols are as follows: For AC18-0177, original approval 03 December 2018, renewed annually, expiry 29 November 2022; for AC18-0191, original approval 17 February 2019, renewed annually, expiry 9 Janary 2023.

Embryos used for RNA-seq were from three independent litters each of the “young” (7–13 weeks of age) and “aged” (43–50 weeks of age) female groups, a strategy pursued to rule out potential litter effects and maximize statistical power. In the young group, one embryo was selected from each litter; for the aged group, we selected two embryos per litter to a total of six conceptuses. All embryos were matched by somite stage, a broadly accurate means of staging embryos. All selected embryos were uniform in size, thus minimizing differences arising purely as a consequence of variation in developmental stage. Dissections of the heart, brain, and facial prominences of E10.5 embryos from timed matings were performed as outlined in the schematic drawing. The placentas were left intact and hence included the maternal decidual portion. All tissue samples were snap frozen in liquid nitrogen and stored at −80 °C. 

### 2.2. RNA Isolation

RNA extractions were performed using the mirVana miRNA isolation kit (ThermoFisher Invitrogen AM1561, Waltham, MA, USA). All isolated RNAs were resuspended in DEPC-H_2_O, quantified using a Nanodrop^TM^ spectrophotometer and quality-verified. RNA samples from the placenta, heart, face, and brain were processed for RNA sequencing using the NEB Ultra II Directional RNA Library Prep kit (E7760) by UCalgary’s Centre for Health Genomics and Informatics facility. RNA sequencing was performed on an Illumina NovaSeq 6000 sequencer using a 50 bp paired-end protocol to a read depth of 14-31M per library (Figure S1). 

### 2.3. RNA Sequencing Analysis (RNA-seq)

Sequencing data were supplied as raw reads in the form of FASTQ files. These files were run through FASTQC to determine the quality of each dataset, and all files were deemed high quality. The FASTQ files were then aligned to the reference mouse genome GRCm38 Primary Assembly (C57BL/6J) (GCF_000000055.19) using the STAR aligner and sorted by coordinate. The number of mappable reads per file was consistently in the range of 90%. After the FASTQ files were aligned to the reference mouse genome, count tables were generated using htseq to determine the number of aligned reads per gene per sample. The reads were counted using the command “intersection-nonempty” to assign each aligned read count to the best matched gene. Since the library generation protocol results in reverse strand reads, the strand directionality was specified in the script to get accurate strand-specific counts. The DESeq2 package in R was used to determine differentially expressed genes using a false discovery rate cutoff of less than 0.1 and a log2fold change cutoff of larger than or equal to 2. The SeqMonk program (https://www.bioinformatics.babraham.ac.uk/projects/seqmonk/, accessed on 12 December 2022) was used to visualize subsets of genes, and to generate heat maps of differentially expressed gene clusters. All these webtools were accessed between January 2021 and November 2022.

### 2.4. Immunofluorescence (IF)

For tissue preparation, placentas and embryos were fixed in 4% paraformaldehyde and processed either for routine paraffin embedding (placentas) or embedded in OCT for cryosectioning (embryos). Placental sections (7 µm) were deparaffinized and rehydrated according to standard protocols. Antigen retrieval was performed on placental sections with 2100 Antigen Retriever. Brain cryosections (10 µm) were warmed for 30 min. The cryo- and paraffin sections were blocked in PBS, 0.1% Tween 20 and 0.5% Bovine Serum Albumin, and incubated with tissue-specific antibody. Placental sections were incubated with antibodies against MCT1 (1:200; Sigma-Aldrich, AB1286-l, St. Louis, MO, USA) and MCT4 (1:100; Sigma-Aldrich AB3314P). Brain sections were incubated with antibodies against Ki67 (1:3000; Abcam, ab15580) ), SOX2 (1:200; R&D Systems AF2018, Minneapolis, MN, USA), PAX6 (1:200; Biolegend 901301, San Diego, CA, USA) and NESTIN (1:200; Abcam ab6142, Cambridge, UK). Detection was carried out with the appropriate AlexaFluor488 or 568-conjugated secondary antibody, respectively (1:300; Thermofisher, Waltham, MA, USA). Nuclear counterstaining was performed with 4′,6-diamidine-2′-phenylindole dihydrochloride (DAPI) (MilliporeSigma, 10236276001, Burlington, MA, USA). 

### 2.5. Ki67 Staining Quantification

*Ki67* staining was quantified using ImageJ (https://imagej.nih.gov; website accessed and program downloaded between January–April 2022). Images were thresholded with the same parameters, and the average size of *Ki67* positive nuclei was divided by the average size of DAPI positive cells. Four to five images were quantified for each of the young and aged cohort brain samples. 

### 2.6. TSC Culture

The wild-type blastocyst-derived TS-Rs26 TSC line (a kind gift of the Rossant lab, Toronto, Canada) was cultured as described previously [21,22]. Briefly, TSCs were grown in standard stem cell conditions (“TS full media”): 20% foetal bovine serum (FBS) (Wisent 098150), 1 mM sodium pyruvate (ThermoFisher Scientific 11360-039, Waltham, MA, USA), 1× Anti-mycotic/Antibiotic (ThermoFisher Scientific 15240-062), 50 µM 2-mercaptoethanol (ThermoFisher Gibco 31350, Waltham, MA, USA), 37.5 ng/mL bFGF (Cambridge Stem Cell Institute, Cambridge, UK), and 1μg/mL heparin in RPMI 1640 with L-Glutamine (ThermoFisher Scientific 21875-034), with 70% of the medium pre-conditioned on mouse embryonic fibroblasts (CM). The medium was changed every two days, and cells passaged before reaching confluency. Trypsinization (0.25% Trypsin/EDTA) was carried out at 37 °C for about 5  min. Differentiation medium (“TS base”) consisted of 20% FBS, 1 mM sodium pyruvate, 1× Anti-mycotic/Antibiotic, 50 μM 2-mercaptoethanol in RPMI 1640 with L-Glutamine. 

### 2.7. Stromal Cell Conditioned Media Effect on Trophoblast Stem Cells

Uterine decidual stromal cells were isolated from young (7–12 wk) and aged (43–50 wk) C57BL/6 females as described previously [18]. Stromal cell conditioned media (STR-CM) was generated by plating approximately 1 × 10^4^ stromal cells in 6-well plates with 2.5 mL of TS base media in the presence of 10 nM 17β-Estradiol (E2, Sigma E2758), 1 µM Medroxyprogesterone 17-acetate (P4, Sigma M1629) and 10µM 8-Bromoadenosine 3′,5′-cyclic monophosphate (cAMP, Sigma B5386) to induce decidualization. This plating strategy achieved a sub-confluent cell density. The media was collected after three days, replenished once, collected again after three days, and pooled into stocks of “young STR-CM” and “aged STR-CM”.

TSCs were plated from sub-confluent cultures at 1:20 splitting ratios into 12-well plates, and three wells each were incubated with (i) 70% young STR-CM, 30% TS base, 37.5 ng/mL bFGF, 1 μg/mL of heparin (Y-STEM); (ii) 70% aged STR-CM, 30% TS base, 37.5 ng/mL bFGF, 1 μg/mL heparin (A-STEM); (iii) 70% young STR-CM and 30% TS base only (Y-DIFF); and (iv) 70% aged STR-CM and 30% TS base only (A-DIFF). After three days, RNA was isolated and stored in a −80 °C freezer. This experimental set-up was repeated three independent times to obtain biological replicate experiments.

### 2.8. Reverse Transcription and PCR (RT-qPCR) Analysis

Total RNA was extracted from cultured cells using TRI reagent (Sigma T9424) according to the manufacturer’s instructions. 1–2 µg of total RNA was used for cDNA synthesis with RevertAid H-Minus reverse transcriptase (Thermo Scientific EP0451) and a mix of oligo-d(T)18 (ThermoFisher FERSO132) and random primers (ThermoFisher FERSO142). Quantitative PCRs (qPCRs) were performed using intron-spanning primer pairs and the QuantiFast (Qiagen 25057) or QuantiNova (Qiagen 208057) SYBR reagent on a ThermoFisher QuantStudio 6 Flex Real-Time PCR System. 

### 2.9. Primer Sequences

Cdx2-FAGTGAGCTGGCTGCCACACTCdx2-RGCTGCTGCTGCTTTCTTCTTGAEsrrb-FAGTACAAGCGACGGCTGGEsrrb-RCCTAGTAGATTCGAGACGATCTTAGTCAEomes-FTCGCTGTGACGGCCTACCAAEomes-RAGGGGAATCCGTGGGAGATGGAGcm1-FACTTCTGGAGGCACGACGGAGcm1-RTCGGGATTTCAGCAGGAAGCGHand1-FGAACTCAAAAAGACGGATGGTGGHand1-RCGCCCAGACTTGCTGAGGMct1/Slc16a1-FCTCCAGTGCTGTGGGCTTGGMct1/Slc16a1-RGCGATGATGAGGATCACGCCAPl1/Prl3d1-FTTATCTTGGCCGCAGATGTGTPl1/Prl3d1-RGGAGTATGGATGGAAGCAGTATGACPlf/Prl2c2-FAACGCAGTCCGGAACGGGGPlf/Prl2c2-RTGTCTAGGCAGCTGATCATGCCASyna-FCCTCACCTCCCAGGCCCCTCSyna-RGGCAGGGAGTTTGCCCACGASynb-FTCCGGAAAGGGACCTGCCCASynb-RCAGCAGTAGTGCGGGGTGCCTpbpa-FACTGGAGTGCCCAGCACAGCTpbpa-RGCAGTTCAGCATCCAACTGCG

## 3. Results

### 3.1. Transcriptomic Analysis of Embryonic Tissues from Young and Aged Females

**Figure 1:**
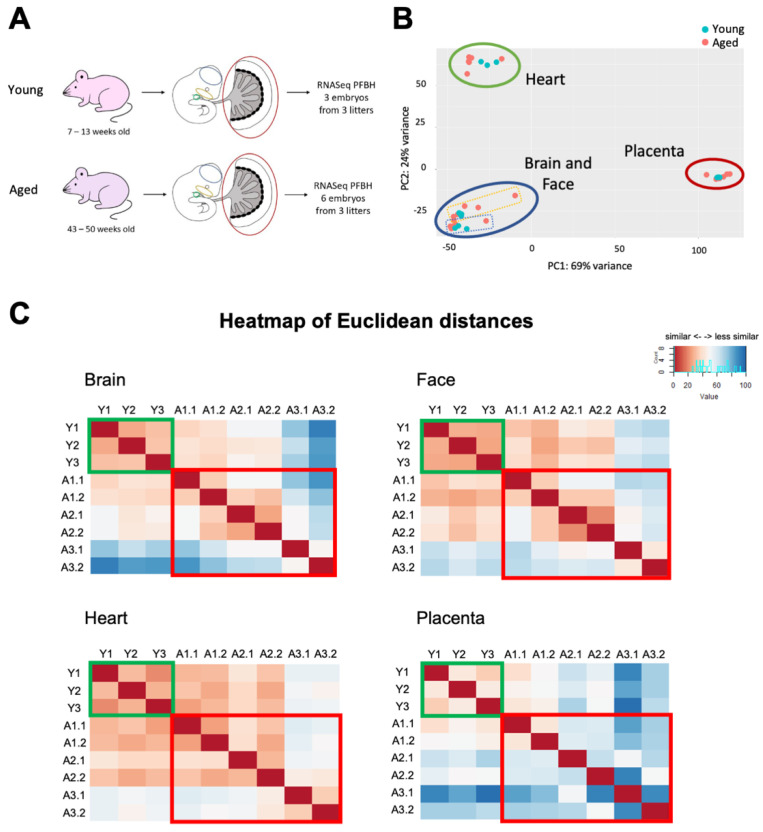
RNA-sequencing analysis of embryonic tissues and placentas from conceptuses developed in young and aged females. (**A**) Experimental design of tissue collection of placenta (P), face (F), brain (B), and heart (H) samples of E10.5 embryos. E10.5 embryos were obtained from young (7–13 weeks) or aged (43–50 weeks) C57BL/6N females. Tissues dissected are encircled and colour-coded: blue for brain, yellow for face, green for heart, and red for placenta. In the young cohort, tissues from 3 embryos were sequenced with one embryo per litter (Y1, Y2, Y3). In the aged cohort, tissues from 6 embryos were sequenced with two embryos per litter to account for increased variability. (**B**) PCA plot of RNA-seq data which clusters samples by tissue type. Circles demarcate samples belonging to the tissues indicated. Dotted yellow and blue rectangles demarcate face and brain samples, respectively. Blue and red dots correspond to tissue samples from young and aged females, respectively. (**C**) Data as in (B) plotted as heatmaps of Euclidean distances per tissue. Dark red indicates higher similarity, while dark blue indicates larger differences. Green and red boxes demarcate samples from young and aged females, respectively. The first and second numbers of sample IDs indicate the litter and embryo numbers, respectively.

To assess the potential influence of AMA on transcriptional regulation in key embryonic tissues, we isolated the brain, facial prominences, heart, and placenta from E10.5 mouse conceptuses of three independent pregnancies in 7–13 weeks (young) or 43–50 weeks (aged) old C57BL/6 females, and subjected them to transcriptomic profiling by RNA sequencing (RNA-seq) (Figure 1A). These four tissues were selected due to their shared sensitivity to co-occurring developmental problems and their high frequency of association to rare and common dysmorphologies in humans [20,23]. All embryos were broadly uniform in size and stage-appropriate in their developmental features (Table 1). We selected one conceptus each from the young pregnancies, and two per litter from the aged pregnancies for a total of 3 “young” and 6 “aged” samples, respectively.

As a first line of investigation, we determined the transcriptional similarity between all four tissue types by principal component analysis (PCA) (Figure 1B). As anticipated, each tissue formed a distinct cluster irrespective of maternal age or fetal sex, with brain and face overlapping in their expression profiles. The heart and placental samples were separated along both PC1 and PC2, illustrating the profound differences in their transcriptional landscapes. In addition to the tissue-type based separation, there was a marked difference in how the samples clustered as a function of maternal age. The samples from young females clustered tightly in comparison to the samples from aged females which had a much wider distribution range (Figure 1B, Figure S2). This was most notable in the brain and face where a few aged samples were substantially distanced from the main tissue-specific cluster. 

### 3.2. Maternal Age Increases the Transcriptional Variability in Embryonic Tissues

To investigate the transcriptional variability within each tissue in more detail, we generated heatmaps of Euclidean distances for the four tissues analyzed, i.e., the brain, face, heart, and placenta (Figure 1C). All samples developed in young mothers exhibited a high degree of similarity within each tissue. By contrast, samples from the aged female cohort showed a much greater extent of variability in gene expression. Among the individual tissue types, it was notable that the embryonic brain displayed the most pronounced transcriptional variation within the samples from aged females, while the heart had the least. The placenta had the most variability overall, regardless of maternal age, in line with the well-known extent of morphological variability that is commonly observed between placentas even in litters of inbred mouse strains [24]. Our transcriptomic data underpin these morphological observations by demonstrating that the placenta is inherently transcriptionally heterogenous even in normal, healthy pregnancies. This contrasts with the three embryonic tissues that are characterized by a much more tightly regulated transcriptional landscape. Therefore, although the placenta exhibited the largest Euclidean distances of gene expression in aged pregnancies, the most significant change in variability occurred in the brains of embryos developed in young versus aged females.

### 3.3. Maternal Age Has a Differential Impact on Brain, Face, Heart, and Placenta 

**Figure 2:**
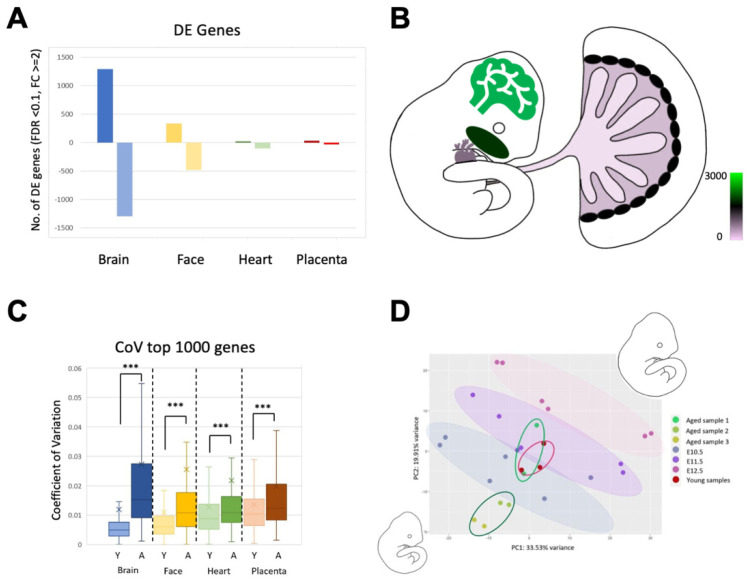
The differential impact of AMA on the brain, face, heart, and placenta. (**A**) Graph depicting the number of differentially expressed genes per tissue comparing samples of conceptuses developed in young vs. aged females. Bars above and below zero indicate up- and down-regulated genes, respectively. (**B**) Schematic diagram pseudo-coloured to depict the differential impact of AMA on the brain, face, heart, and placenta based on the number of differentially expressed genes. The colour scheme reflects number of differential genes. Brain is the most affected by maternal age while placenta appears the least affected, in part due to the intrinsic higher transcriptional variability in this organ. (**C**) Coefficient of variation of the top 1000 most highly expressed genes across tissues. Y and A stands for samples from young and aged females, respectively. Three replicates were used for young samples, while six replicates were used for aged samples. Bar colours are coordinated to tissue type with blue for brain, yellow for face, green for heart, and red for placenta. The “x” indicates the sample mean. Error bars indicate standard deviation. Pair-wise comparisons between samples of the same tissue type were performed using a two-tailed Student’s *t*-test. *** *p* < 0.001. (**D**) PCA plot of RNA expression data across a developmental timeline for embryonic brain. RNA-Seq data [26] was used to generate a timeline of E10.5–12.5 which is indicated by the coloured ovals. Brain samples from the young litters of our study are circled in red. Brain samples from aged litter 1 are circled in bright green, while brain samples from aged litters 2 and 3 are circled in dark green.

The detection of an increased transcriptional dissimilarity in tissues of embryos conceived in aged females prompted us to further explore the impact of AMA on the brain, face, heart, and placenta. Therefore, we first determined the number of differentially expressed (DE) genes using DESeq2 for each tissue depending on maternal age (Figure 2A). In keeping with expectation from the above observations, the brain had the highest number of DE genes with 1294 up- and 1294-down-regulated genes between samples from young vs. aged females. The number of DE genes was markedly lower in the other tissues, with a total of 814 genes in the face, 123 in the heart, and 61 in the placenta (Figure 2B). We also performed an e-karyotyping analysis on the RNA-seq data using a piecewise constant fit algorithm which did not detect any gross chromosomal imbalances [25].

To further examine the tissue-specific transcriptional heterogeneity, we determined the coefficient of variation (CoV) for the top 1000 most highly expressed genes in each tissue of the young and aged cohorts (Figure 2C). Strikingly, the brain tissues from the aged cohort had the highest variability among all samples. This result underscored the heatmaps of Euclidean distances, but further revealed the remarkable magnitude of transcriptional variability in the developing brain as a consequence of AMA. The face had the second-highest CoV in the aged cohort, even if these values were much smaller compared to brain. The heart and placenta had the lowest CoVs of all tissues from the aged cohort. In contrast, the opposite pattern was seen when assessing the tissues from the young cohort: Here, placental samples from young females had the highest coefficient of variation, while the embryonic brain samples from young females had the lowest. These data are fully in line with the Euclidean distance maps (Figure 1C) and further exemplify that the placenta is intrinsically the most variable organ, while the embryonic brain’s transcriptional landscape is normally very tightly regulated, but is most susceptible to developmental dysregulation in pregnancies of AMA. 

### 3.4. AMA Preferentially Affects Embryonic Brain Development

To exclude the possibility that the increased transcriptional heterogeneity in the aged sample cohort was caused by developmental stage-specific variations, we integrated our RNA-seq data with previously published time-course data of staged embryonic brain, heart and face samples at E10.5, E11.5 and E12.5 [26]. Focussing on the brain, the resultant PCA graph highlighted the distinct developmental timepoints from E10.5–E12.5 based on transcriptional signatures (Figure 2D). When assessing our RNA-seq data of the embryonic brain samples from young and aged females in this context, we observed a certain litter effect in embryos from the aged pregnancies (Figure 2D). Thus, while our young control brains overlapped closely with the E10.5–E11.5 range as expected, the aged litters displayed significant differences: The two brain samples from aged litter 1 also clustered around E10.5–E11.5, but the transcriptional profiles of brains from aged litters 2 and 3 mapped into regions of the PCA plot indicative of an earlier developmental stage. This suggests that the expression profiles of embryonic brain samples from aged litters 2 and 3 exhibit signs of a slight developmental delay, while the brain samples from aged litter 1 do not. Intriguingly, this distribution correlates with the fact that the females that produced aged litters 2 and 3 were older than the female that produced aged litter 1 (Table 1; 43 weeks for aged litter 1 versus 50 and 46 weeks for aged litters 2 and 3, respectively). Importantly, when overlaying developmental stage-specific RNA-seq data for heart and face, we did not observe the same developmental delay (Figure S3). This indicates that the brain is particularly sensitive to transcriptional dysregulation as a function of AMA and displays a more aberrant expression signature that is indicative of a developmental delay where this is not evident in other embryonic tissues.

### 3.5. Neurodevelopmental Genes Are Profoundly Affected by AMA

**Figure 3:**
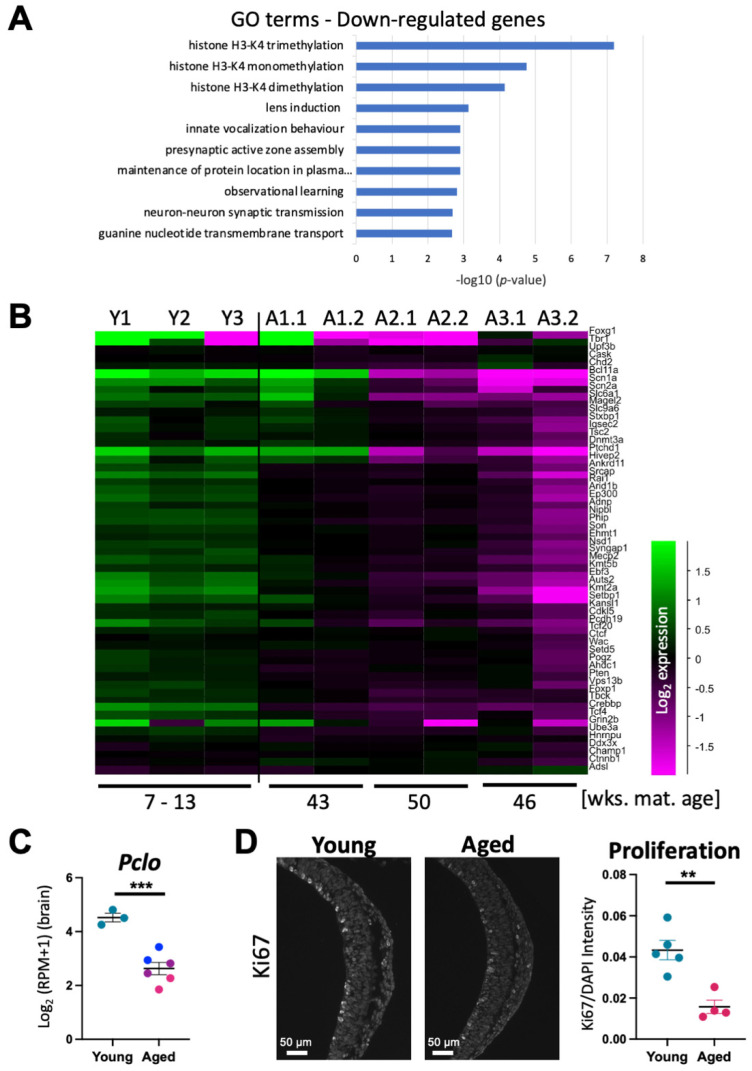
Key genes are down-regulated in embryonic brain samples from aged females. (**A**) Gene ontology (GO) terms associated with the most down-regulated genes in aged brain samples determined using Panther GO analysis. (**B**) Heatmap displaying gene expression levels of high confidence neurodevelopmental disorder genes from Leblond et al. 2021 [27]. Brain samples from pregnancies in young females are on the left and those from pregnancies in aged females on the right, with the first and second number indicating litter and embryo number, respectively. Maternal age in weeks is shown on the bottom. Scale is log_2_ fold change. (**C**) Log_2_-transformed reads per million (RPM+1) of *Pclo*, a gene involved in schizophrenia and bipolar disorder, in young vs. aged cohort brains. Blue, purple, and pink points in the aged cohort column correspond to samples from aged litters 1, 2, and 3, respectively. Error bars indicate SEM. Statistical analysis was performed using an unpaired, two-tailed Student’s *t*-test. *** *p* < 0.001 (**D**) Immunofluorescence of Ki67 in forebrain samples of E10.5 embryos from young and aged females. Quantification was performed by calculating Ki67 intensity relative to nuclear area determined by DAPI staining. Error bars indicate SEM. Statistical analysis was performed using an unpaired, two-tailed Student’s *t*-test. ** *p* < 0.01.

With the consistent result that the expression profiles of embryonic brains were the most perturbed by AMA, we investigated which biological processes were affected in this tissue. Using Panther Gene Ontology (GO) analysis, we found that the down-regulated genes were enriched for terms related to transcriptional regulation (Figure 3A). Specifically, histone H3-K4 methylation was the most significant pathway for down-regulated genes, suggesting an epigenetic basis of the wide-spread transcriptional de-regulation in these samples. In addition, the down-regulated genes were enriched for neuronal-specific terms such as lens induction, presynaptic active zone assembly, and observational learning. By contrast, terms associated with the up-regulated genes were centred around ribosomes and RNA modifications, which is overall not as relevant or specific to brain function directly (Figure S4A).

AMA is positively correlated with an enhanced frequency and risk of neurodevelopmental disorders. To better understand how neurodevelopmental genes are affected by maternal age, we assessed the expression patterns of a cohort of high confidence neurodevelopmental disorder genes (HC-NDD) in the brain samples from aged vs. young pregnancies [27]. Remarkably, all HC-NDD genes were consistently down-regulated in the embryonic brains from the aged cohort (Figure 3B). Importantly, this was evident even in the samples of litter 1 that did not display an overt developmental delay-associated expression signature. One example of this was the gene encoding the presynaptic cytomatrix protein Piccolo (*Pclo*), which is associated with schizophrenia and bipolar disorder [28]. *Pclo* was significantly down-regulated in all brain samples from the aged cohort (Figure 3C). Moreover, there was a clear trend that this down-regulation became more pronounced as maternal age advanced further. 

To further evaluate whether the transcriptional differences observed translated into gross anatomical changes, we stained E10.5 embryos from young and aged litters for the neural stem and progenitor markers SOX2 and NESTIN, and for the early radial glial cell marker PAX6. No overt changes in the distribution or intensities of these markers were observed between the brains of embryos from young and aged females. However, staining for the proliferation marker Ki67 revealed a significantly reduced rate of proliferation in the aged samples, as determined by the area of Ki67 fluorescence intensity over total area of nuclei. The ratio of Ki67-positive cells was decreased by a factor of 2.6× in the aged cohort brain samples (Figure 3D). However, the number of nuclei in midbrain sections did not differ between the young and aged cohorts. This indicates that although expression of a proliferation marker was decreased, the number of nuclei had not been affected at this stage of development.

### 3.6. AMA Perturbs the Trophoblast Compartment of the Placenta

**Figure 4:**
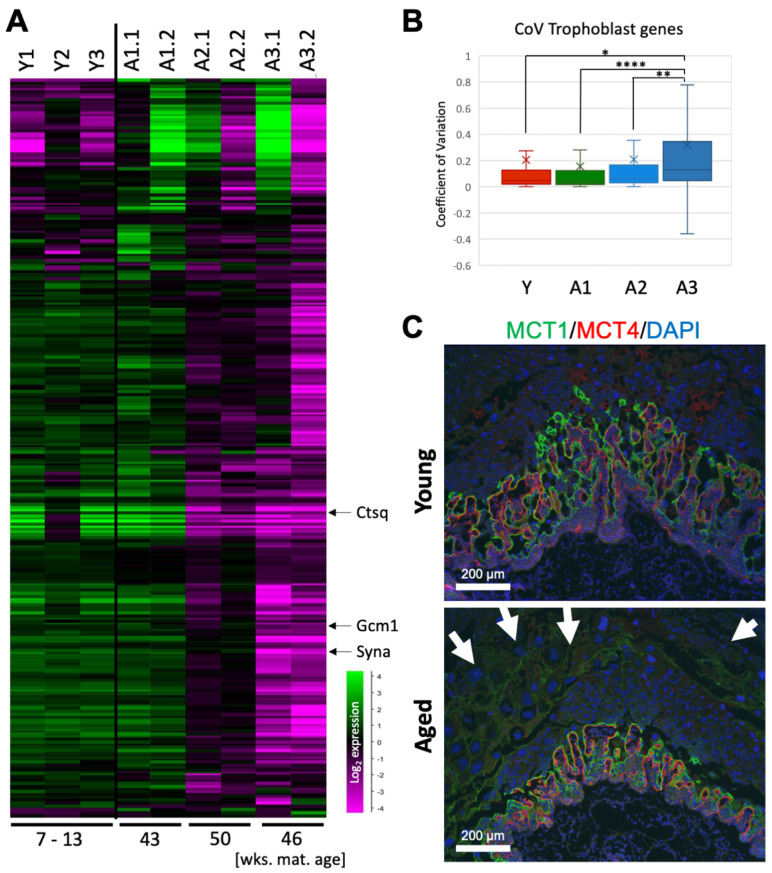
Impact of AMA on the trophoblast compartment of the placenta. (**A**) Heatmap depicting gene expression levels of trophoblast-enriched genes in placental samples. Key trophoblast marker genes are indicated. *Gcm1* and *Syna* are syncytiotrophoblast markers while *Ctsq* is a sinusoidal trophoblast giant cell marker. Maternal age in weeks is shown on the bottom. (**B**) Coefficient of variation for trophoblast-enriched genes in the placenta of young an aged samples. Left to right: young litters, aged litter 1, aged litter 2, aged litter 3. The young litter group has 3 biological replicates, while aged litters 1, 2, and 3 have two replicates each. The “x” indicates the mean. Error bars indicate standard deviation. Statistical analysis was performed using a one-way ANOVA with Bonferroni’s post hoc test. * *p* < 0.05, ** *p* < 0.01, **** *p* < 0.0001. (**C**) Double immunofluorescence for MCT1 and MCT4, demarcating the syncytiotrophoblast layers I and II, respectively, in the mouse placental labyrinth. The fetal portion of the placenta and the labyrinth in particular tend to be under-developed in conceptuses to aged females. Additionally, obvious are vast numbers of trophoblast giant cells (arrows) in the aged samples.

The placenta forms the interface between the mother and the fetus and constitutes the point of contact with the aging maternal environment. Therefore, we reasoned that any effects of maternal age on the embryo must be mediated through the placenta. Taking our transcriptomics approach to compare gene expression in placentas developed in young and aged females, we specifically assessed the expression of genes characteristic of the fetal portion of the placenta which may be affected as a direct consequence of decidual dysfunction [18]. This analysis identified significantly reduced expression levels of many trophoblast genes in the placental samples from aged litters 2 and 3 which included, for example, the syncytiotrophoblast markers *Gcm1* and *Syna*, and the sinusoidal giant cell marker *Ctsq* (Figure 4A). These data are in line with previous observations [18]. By comparison, placentas of aged litter 1 were not, or only mildly, affected. These data demonstrate that for both embryonic and placental tissues, a significant shift is observed towards more dysregulated gene expression patterns within a rather narrow window of maternal age, i.e., between approximately 43 and 46–50 weeks of age. 

Due to this “cliff-edge” effect, we repeated the differential gene expression analysis after excluding samples from aged litter 1, which resulted in a substantial increase in the number of DE genes per tissue based on maternal age, as well as in an increase in the number of overlapping DE genes across all tissue types (Figure S4B–D). We also assessed CoVs specifically for trophoblast-enriched genes (Figure 4B). The only significant differences in variability occurred with the placental samples from aged litter 3. Their variability in trophoblast-specific gene expression was significantly greater than that of the other litters which had very similar CoVs. This illustrates that the embryos that developed in one of the older females had the highest amount of transcriptional variability in the trophoblast, which could lead to more severe embryonic outcomes, such as a higher risk of neurodevelopmental disorders. 

To further evaluate the functional impact of this transcriptomic dysregulation, we stained a separate set of E10.5 placentas for the syncytiotrophoblast markers MCT1 and MCT4, which demarcate the feto-maternal exchange surface of the placental labyrinth [20]. These stainings clearly revealed that the fetal portions of placentas from aged litters were consistently smaller with a poorer developed and less expanded vascular area at this stage (Figure 4C).

### 3.7. Aged Uterine Stromal-Cell Conditioned Media Accelerates Differentiation of Trophoblast Stem Cells

**Figure 5:**
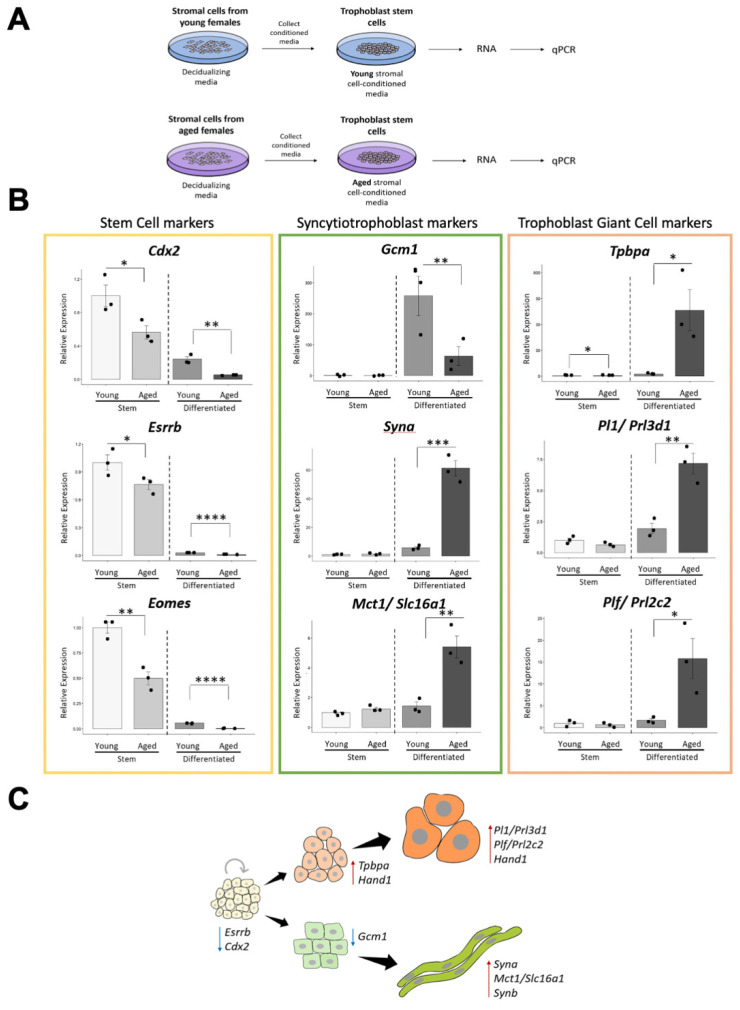
Aged decidual stromal-cell conditioned media accelerates differentiation of trophoblast stem cells. (**A**) Experimental set-up to explore the effect of uterine stromal cell conditioned medium obtained from cells of young vs. aged females on trophoblast stem cells. (**B**) RT-qPCR data of trophoblast cell-type specific marker genes upon exposure to young vs. aged decidual stromal-cell conditioned media. Cells were assessed in stem cell conditions and after three days of differentiation. Genes assessed are colour-coded for stem cell (yellow), syncytiotrophoblast (green) and trophoblast giant cell (orange) markers, respectively. Data are normalized to the stem cell conditions exposed to young conditioned media and plotted as mean +/− SEM. Data are representative of three independent biological replicates. Statistical analysis was performed using a two-tailed Student’s *t*-test. * *p* < 0.05, ** *p* < 0.01, *** *p* < 0.001, **** *p* < 0.0001. (**C**) Schematic depicting the aged decidual stromal-cell effect on trophoblast stem cells. Trophoblast stem cells differentiate more rapidly into the giant cell and syncytiotrophoblast lineages and precociously exit the stem cell state. Red and blue arrows indicate up- and down-regulation of genes, respectively.

Trophoblast cells are the extra-embryonic cells in direct contact with the aging maternal uterine environment. Signals from the decidualizing uterine environment are known to be of key importance for normal placental development [18,29,30]. Therefore, we next sought to assess the direct impact of uterine decidualizing stromal cells on trophoblast differentiation as a function of maternal age. To this end, we used trophoblast stem cells (TSCs) as a well-established tool to model early placental development, and exposed them to medium conditioned by decidualizing stromal cells (STR-CM) isolated from uteri of either young or aged females (Figure 5A). Our data showed that the STR-CM from aged females resulted in reduced levels of stem cell markers *Eomes*, *Cdx2* and *Esrrb*, and syncytiotrophoblast precursor marker, *Gcm1*, which peaks early on in differentiation. By contrast, aged STR-CM caused an elevated expression of spongiotrophoblast and trophoblast giant cell markers *Tpbpa*, *Prl2c2, Prl3d1,* and *Hand1,* and syncytiotrophoblast markers *Mct1/Slc16a1, Syna,* and *Synb*, compared to the effect of STR-CM from young females (Figure 5B,C, Figure S5). These data indicated that the STR-CM from aged females induced TSCs to precociously exit the stem cell state and to differentiate more rapidly into both the giant cell and syncytiotrophoblast lineages. This differentiation-promoting effect of aged stromal cells closely resembled the phenotype of placentas developed in AMA females, which exhibit a smaller fetal trophoblast compartment indicative of decreased progenitor cell expansion and in particular a reduction of the placental labyrinth (Figure 4A,C). Interestingly, the parietal giant cells at the feto-maternal interface are also increased in numbers in placentas from the aged cohort (Figure 4C, arrows), again fully reflecting the aged STR-CM effect we see in vitro. Thus, the decidualizing uterine environment of aged females induces placental trophoblast defects that in turn are likely the root cause of the observed transcriptional dysregulations in embryonic tissues, most notably affecting the developing brain.

## 4. Discussion 

AMA, specifically at first pregnancy, is becoming increasingly prevalent in our society and introduces a higher incidence of pregnancy complications and embryonic abnormalities. In the current study, we aimed to investigate how AMA impacts the transcriptional landscapes of the placenta, heart, brain, and face in overtly normal pregnancies, thus uncovering effects that do not have a karyotypic basis as a result of meiotic defects in the oocyte. Overall, we found that embryonic tissues display transcriptomic changes as a function of maternal age even in the absence of morphological abnormalities in the embryo itself. 

During early development, the heart and the placenta are the first two organs to form from around E8.5 onwards. Despite this developmental timing, we saw the strongest effect of AMA on embryonic brain development, coinciding temporally with the stage when placental function becomes critical at mid-gestation and developmental progression relies on nutrient transfer capacity across the placental labyrinth. This bottleneck likely explains the profound effects we observe on brain development, perhaps as a result of subtle defects or developmental delays in the trophoblast compartment of the developing placenta. 

This conclusion is underscored by the brain exhibiting the largest increase in transcriptional heterogeneity due to AMA. Although the embryonic face and heart also showed a significant increase in transcriptional variability, this dysregulation did not reach the same magnitude as the brain. An enhanced transcriptional heterogeneity can result in a higher incidence of various diseases, such as heart disease, cancer, and stroke, increased inflammation, and disruptions to cellular proliferation and metabolism [31,32]. Therefore, the marked increase in transcriptional variability that we observe in embryonic tissues as a function of AMA may have detrimental consequences on the normal progression of development, but specifically on neurodevelopment.

When exploring the set of consistently de-regulated genes for functional pathway enrichments, the term ‘cognition’ was identified in the observational learning category amongst the down-regulated genes in brain. This finding implies that many genes important for proper brain function exhibit decreased expression levels at mid-gestation in the aged cohort. To further investigate the significance of the profoundly down-regulated genes in brains of the aged cohort, we used a HC-NDD gene list and found a pronounced dysregulation of many of these neurodevelopmental genes specifically. The neurodevelopmental disorders on which this gene list is based include intellectual disability, learning disorders, ADHD, and autism spectrum disorder (ASD) [27]. Previous studies have found associations between AMA and ASD, as well as between AMA and schizophrenia [33,34,35]. Therefore, our data show that brains of mid-gestation mouse embryos with an overtly normal appearance exhibit perturbed gene expression signatures due to AMA that will undoubtedly increase the risk of manifesting themselves in postnatal neurodevelopmental disorders. Future studies will have to explore whether perhaps AMA also introduces a sex-specific effect on fetal development that may have lifelong health implications.

To determine if the transcriptional differences in the embryonic brains of conceptuses developed in AMA females coincide with morphological differences early on, we found that the proliferation marker Ki67 was decreased almost 3-fold in E10.5 brain samples from the aged cohort. Although this marked decreased in Ki67 did not alter cell numbers in the brains from the aged cohort yet, lower proliferation rates coupled with the marked transcriptional dysregulation has the potential to impact further brain development as gestation progresses. 

Transcriptional noise has been shown to increase with age in many tissues and individual cell populations [31,32,36,37,38,39,40,41,42]. What is remarkable in our study, however, is that we observe such an increase in transcriptional variability in embryonic tissues that are themselves not aged, but only indirectly exposed to an aging uterine environment of the mother. Thus, cell biological aspects revealed in “classical” aging studies such as accumulations of somatic DNA damage or somatic mutations can likely be ruled out as a source of an aging phenotype in our study. Furthermore, the recurrence of the same phenotypes and de-regulated pathways argues against sporadic, random chromosomal abnormalities. Rather, they point to conserved defects that occur as a consequence of maternal aging and hence will play out at the feto-maternal interface, i.e., the placenta. This conclusion is corroborated by the fact that the organ systems at higher risk of developmental defects in mothers of AMA are the exact same ones that are also correlated with placentation defects [12,14,15,20,34,35].

To further explore how an aging uterine environment may affect placental development, we employed an in vitro model to study the impact of endometrial stromal cells on trophoblast (stem) cell behaviour. Our data show that uterine stromal cells, which in vivo are in direct contact with trophoblast, are impacted by AMA such that aged stromal cells induce TSCs to differentiate precociously and at an accelerated rate. These differentiation-promoting effects deplete the trophoblast stem or progenitor cell pool, resulting in the underdeveloped trophoblast portion of the placenta that we commonly observed in conceptuses developed in aging females [20]. The developmental connection between the placenta and the brain, known as the placenta-brain axis, is well documented [43]. Many neuro-behavioural disorders likely arise from placental abnormalities, and neurotransmitters such as serotonin arise solely from the placenta during the mid-gestational time window that is critical for neurodevelopment [44,45,46,47,48,49]. Therefore, our data are consistent with the fact that placental defects preferentially impact embryonic brain development. Moreover, we pin the source of the placental defects down to deficits in the decidualizing uterine stroma.

Taken together, here we show that AMA impacts embryonic tissue expression profiles even in embryos that have developed overtly normally. This effect is most profoundly observed in the mid-gestation brain, i.e., during a key critical time period for neurodevelopment. These common transcriptomic changes are likely mediated through the placenta, whose trophoblast portion is impeded in normal expansion and differentiation through defective signaling cues emanating from the aged uterine stroma. Even if many of these mid-gestational differences may be mitigated during later development to produce healthy offspring, these data highlight the increased risk of maternal age on reproductive outcome, with neurodevelopment being the most sensitive to such early perturbations and with potential for lifelong impact.

## Figures and Tables

**Table 1:** Details of litters and embryos used.

Litter	Mat. Age [wks]	No Implantation Sites	Embryo ID	Somite Number *	Sex
**Young Litter 1**	13	9	**Y1**	N.D.	MALE
**Young Litter 2**	7	9	**Y2**	N.D.	MALE
**Young Litter 3**	8	9	**Y3**	N.D.	FEMALE
**Aged Litter 1**	43	9	**A1.1**	N.D.	FEMALE
			**A1.2**	N.D.	MALE
**Aged Litter 2**	50	11	**A2.1**	N.D.	MALE
			**A2.2**	24	MALE
**Aged Litter 3**	46	6	**A3.1**	21	MALE
			**A3.2**	24	FEMALE

* All embryos were developed appropriately for their developmental stage. In the interest of time spent during dissection to preserve tissue integrity for optimal RNA quality, somite counts were not performed in every instance.

## Data Availability

Sequence data were deposited under accession GSE209522.

## Supplementary Materials

The following supporting information can be downloaded at: https://www.mdpi.com/article/10.3390/cells12010076/s1, Figure S1: RNA-seq metrics; Figure S2: Increased transcriptional variability as a functions of AMA by tissue type; Figure S3: Developmental time course integration of heart and brain samples; Figure S4: Differential gene expression analysis; Figure S5: Impact on trophoblast cell types-specific gene expression as a consequence of uterine stromal cell conditioned medium from young and aged females.
