# Supplementary material for: Advanced Maternal Age Differentially Affects Embryonic Tissues with the Most Severe Impact on the Developing Brain
Source: Cells. 2022 Dec 24;12(1):76. doi: 10.3390/cells12010076 (PMC9818809; doi:10.3390/cells12010076)
Supplement: Supplementary file 1 [file cells-12-00076-s001.zip › cells-2070302-supplementary.pdf]

Supplementary Materials

Advanced maternal age differentially affects embryonic tissues with the most severe impact on the developing brain

Caroline Kokorudz[1], Bethany N. Radford[1], Wendy Dean[2,3]* and Myriam Hemberger[1,3]*

Figure S1

A Young

Sample	# of reads	# of mappable reads	# mappable reads/tissue
B5-1 L001	18115368	16405809 **(90.56%)**	16736193
B5-1 L002	18202445	16489012 **(90.59%)**	
B6-2 L001	15498559	13939001 **(89.94%)**	
B6-2 L002	15607380	14046066 **(90.00%)**	
B7-5 L001	21867857	19699874 **(90.09%)**	
B7-5 L002	22010132	19837401 **(90.13%)**	
F5-1 L001	18269895	16462042 **(90.10%)**	17991353
F5-1 L002	18355323	16545200 **(90.14%)**	
F6-2 L001	19816443	17705112 **(89.35%)**	
F6-2 L002	19916812	17801554 **(89.38%)**	
F7-5 L001	21843315	19663937 **(90.02%)**	
F7-5 L002	21947617	19770277 **(90.08%)**	
H5-1 L001	21937313	20033574 **(91.32%)**	16809203
H5-1 L002	22065962	20160279 **(91.36%)**	
H6-2 L001	14553119	13009485 **(89.39%)**	
H6-2 L002	14627585	13085492 **(89.46%)**	
H7-5 L001	19003397	17203426 **(90.53%)**	
H7-5 L002	19164522	17362964 **(90.60%)**	
P5-1 L001	21667487	19390523 **(89.49%)**	17234359
P5-1 L002	21812585	19535173 **(89.56%)**	
P6-2 L001	17140098	15062724 **(87.88%)**	
P6-2 L002	17220401	15145847 **(87.95%)**	
P7-5 L001	19003152	17072871 **(89.84%)**	
P7-5 L002	19134723	17199016 **(89.88%)**	

B Aged

Sample	# of reads	# of mappable reads	# mappable reads/tissue
B_A1-1 L001	25085236	22426812 **(89.40%)**	18930423
B_A1-1 L002	24665766	22034455 **(89.33%)**	
B_A1-4 L001	24658959	21793710 **(88.38%)**	
B_A1-4 L002	24310882	21473392 **(88.33%)**	
B_A2-1 L001	22118628	19428881 **(87.84%)**	
B_A2-1 L002	21659873	19013685 **(87.78%)**	
B_A2-2 L001	18342874	15939254 **(86.90%)**	
B_A2-2 L002	18034015	15658297 **(86.83%)**	
B_A3-1 L001	23250012	20152905 **(86.68%)**	
B_A3-1 L002	22927608	19861151 **(86.63%)**	
B_A3-2 L001	18375294	14848125 **(80.80%)**	
B_A3-2 L002	17990672	14534403 **(80.79%)**	
F_A1-1 L001	19561818	17426617 **(89.08%)**	16731799
F_A1-1 L002	19096899	17001401 **(89.03%)**	
F_A1-4 L001	21502939	19039307 **(88.54%)**	
F_A1-4 L002	21100549	18670659 **(88.48%)**	
F_A2-1 L001	19251902	16925613 **(87.92%)**	
F_A2-1 L002	18931199	16636105 **(87.88%)**	
F_A2-2 L001	19570215	17287715 **(88.34%)**	
F_A2-2 L002	19243009	16988142 **(88.28%)**	
F_A3-1 L001	17463910	15162001 **(86.82%)**	
F_A3-1 L002	17109047	14841656 **(86.75%)**	
F_A3-2 L001	17954439	15571576 **(86.73%)**	
F_A3-2 L002	17572531	15230797 **(86.67%)**	
H_A1-1 L001	20640736	17832644 **(86.40%)**	16545717
H_A1-1 L002	20312012	17537905 **(86.34%)**	
H_A1-4 L001	22410551	19911031 **(88.85%)**	
H_A1-4 L002	21981520	19514867 **(88.78%)**	
H_A2-1 L001	17963091	15958566 **(88.84%)**	
H_A2-1 L002	17571961	15596072 **(88.76%)**	
H_A2-2 L001	17311214	15575568 **(89.97%)**	
H_A2-2 L002	16758893	15063762 **(89.89%)**	
H_A3-1 L001	17565877	15389820 **(87.61%)**	
H_A3-1 L002	17173086	15037790 **(87.57%)**	
H_A3-2 L001	18065976	15871622 **(87.85%)**	
H_A3-2 L002	17655000	15498961 **(87.79%)**	
P_A1-1 L001	21530883	18858378 **(87.59%)**	18973167
P_A1-1 L002	21057867	18429102 **(87.52%)**	
P_A1-4 L001	23323075	20084292 **(86.11%)**	
P_A1-4 L002	22873048	19678823 **(86.03%)**	
P_A2-1 L001	16119215	14339185 **(88.96%)**	
P_A2-1 L002	15796336	14038858 **(88.87%)**	
P_A2-2 L001	18205932	16068119 **(88.26%)**	
P_A2-2 L002	17817190	15712057 **(88.18%)**	
P_A3-1 L001	18314309	14548534 **(79.44%)**	
P_A3-1 L002	17888546	14193807 **(79.35%)**	
P_A3-2 L001	34604859	31198764 **(90.16%)**	
P_A3-2 L002	33879666	30528083 **(90.11%)**	

Figure S1. RNA-seq metrics.
(A) Number of mappable reads per tissue for all young tissues samples RNA-sequenced. B, F, H, and P stand for brain, face, face, and placenta, respectively. L001 and L002 correspond to lanes 1 and 2, respectively. The percentage of mappable reads per sample is bolded. **(B)** Number of mappable reads per tissue for all aged tissues samples RNA-sequenced. B, F, H, and P stand for brain, face, face, and placenta, respectively. L001 and L002 correspond to lanes 1 and 2, respectively. The percentage of mappable reads per sample is bolded.

Figure S2

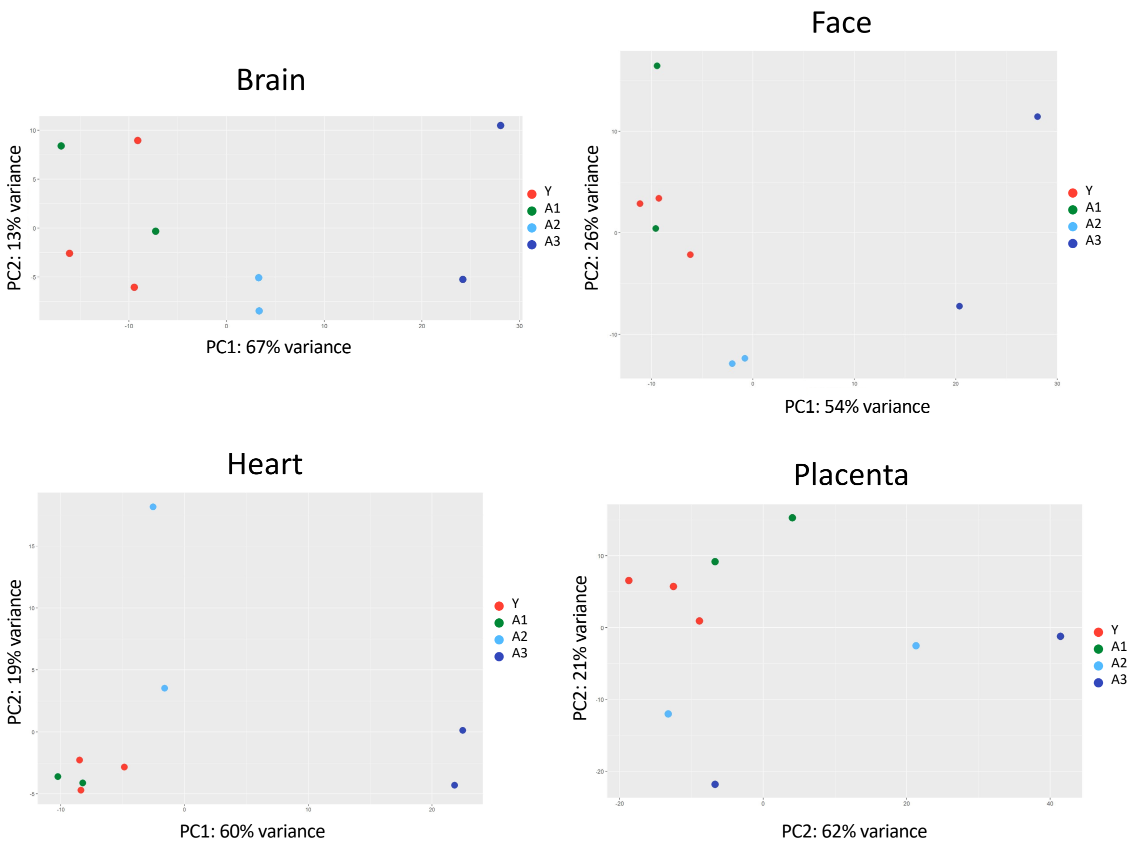

Figure S2. Increased transcriptional variability as a functions of AMA by tissue type.
PCA plots of RNA-seq data clustering samples by tissue type. Top left to bottom right: brain, face, heart, and placental PCA plots. Red, green, light blue, and dark blue dots correspond to tissue samples from young litters, aged litter 1, aged litter 2, aged litter 3, respectively.

Figure S3

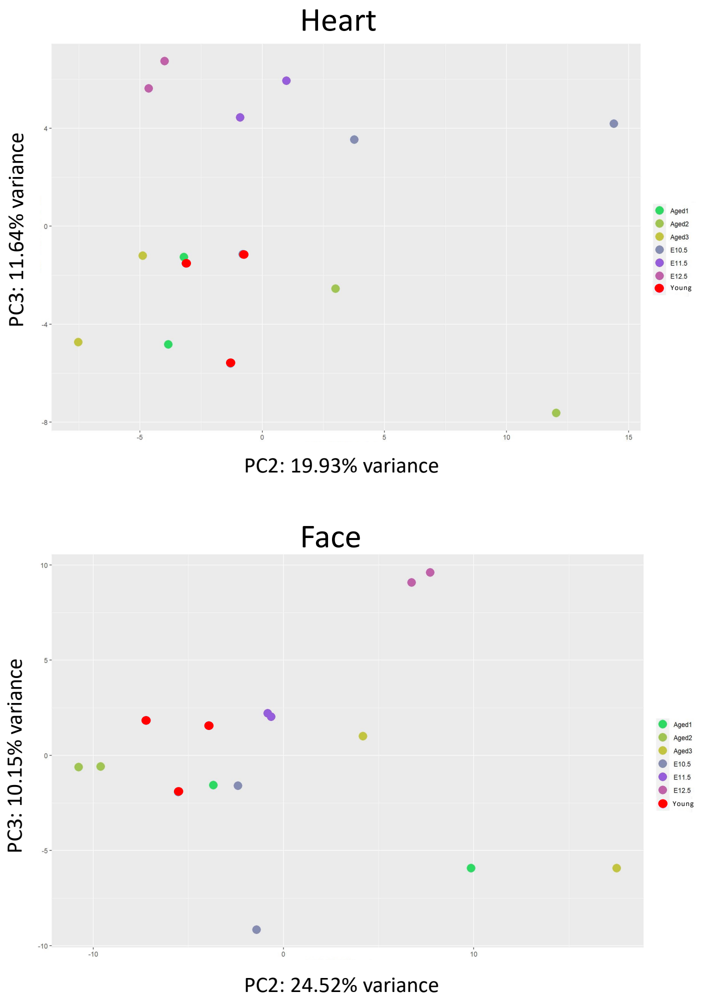

Figure S3. Developmental time course integration of heart and brain samples.
PCAs plot of embryonic heart (top) and brain (bottom) data from the young and aged cohorts, mapped onto a developmental time course of these tissues from E10.5, E11.5, and E12.5. Samples from aged litter 1, 2, and 3 are bright green, green, and yellow, respectively. Samples from the young cohort are red.

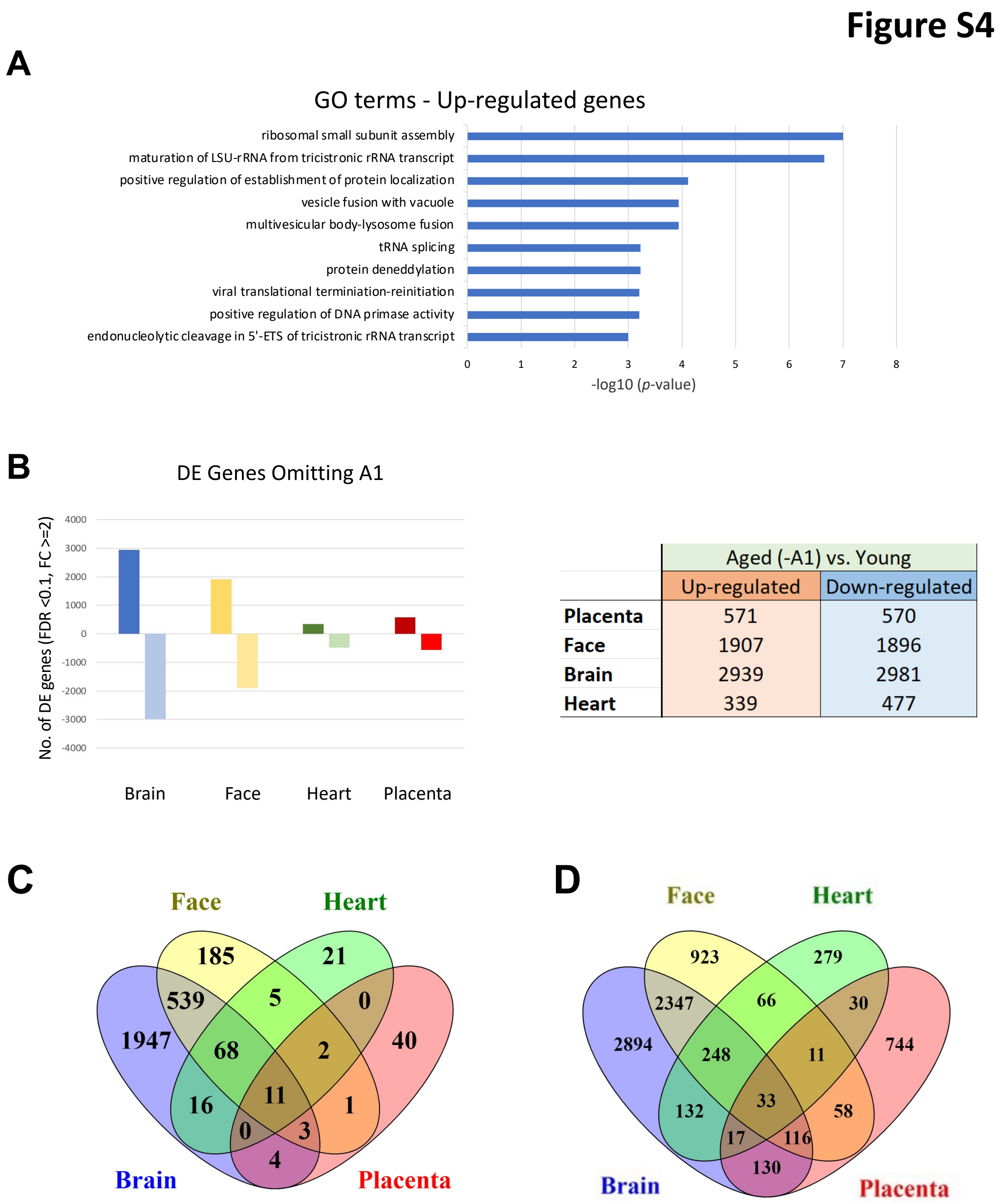

	Aged (-A1) vs. Young	
	Up-regulated	Down-regulated
**Placenta**	571	570
**Face**	1907	1896
**Brain**	2939	2981
**Heart**	339	477

Figure S4. Differential gene expression analysis.
(A) Gene ontology (GO) terms associated with the top up-regulated genes in aged brain samples using Panther GO analysis. **(B)** Number of differentially expressed genes per tissue comparing young vs. aged samples when aged litter 1 is omitted shown in a bar graph (left) and in a table (right). Left: Bars above and below zero indicate up- and down-regulated genes, respectively. **(C)** Number of overlapping up- or down-regulated genes between the face, heart, brain, and placenta. **(D)** Number of overlapping up- or down-regulated genes between the face, heart, brain, and placenta when aged litter 1 is omitted.

Figure S5

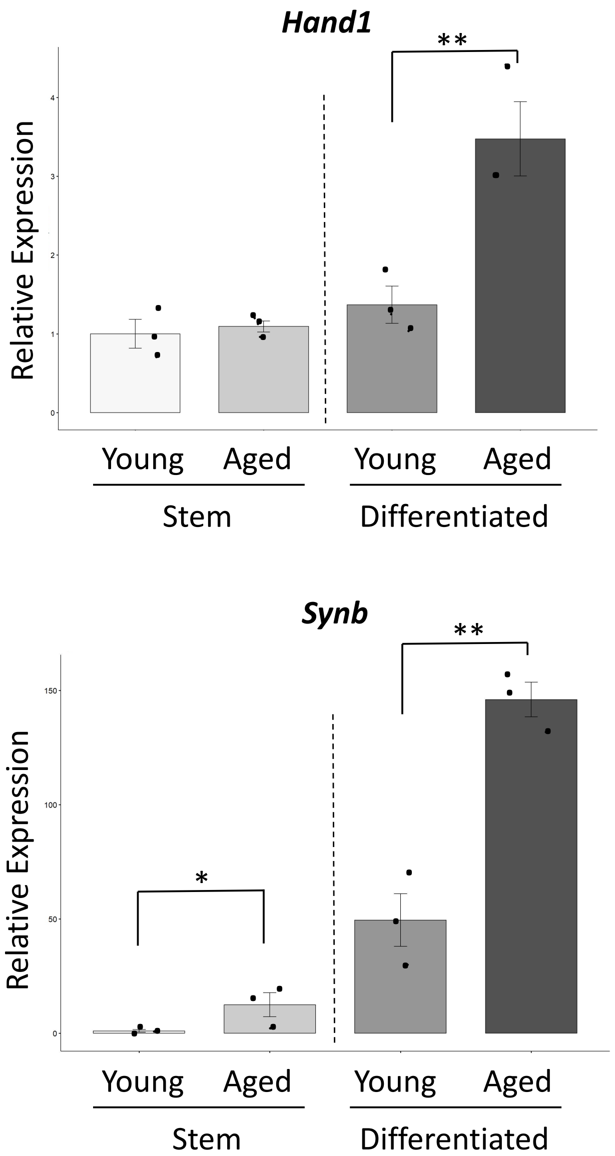

Figure S5. Impact on trophoblast cell types-specific gene expression as a consequence of uterine stromal cell conditioned medium from young and aged females.

RT-qPCR data of trophoblast cell-type specific marker genes on exposure to young vs. aged decidualizing uterine stromal-cell conditioned medium. Cells were assessed in stem cell conditions and after three days of differentiation. *Hand1* is a giant cell marker, while *Synb* is a syncytiotrophoblast marker. Data are normalized to the stem cell conditions exposed to young conditioned media and plotted as mean +/- SEM. Data are representative of three independent biological replicates. *p<0.05, **p<0.01.
